# Stink Bug Feeding Induces Fluorescence in Developing Cotton Bolls

**DOI:** 10.1186/1754-1611-5-11

**Published:** 2011-08-04

**Authors:** Jinjun Xia, Adnan Mustafic, Michael D Toews, Mark A Haidekker

**Affiliations:** 1 University of Georgia, Faculty of Engineering, Athens, GA 30602-4435, USA; 2 University of Georgia, Department of Entomology, Tifton, GA 31793-0748, USA

## Abstract

**Background:**

Stink bugs (Hemiptera: Pentatomidae) comprise a critically important insect pest complex affecting 12 major crops worldwide including cotton. In the US, stink bug damage to developing cotton bolls causes boll abscission, lint staining, reduced fiber quality, and reduced yields with estimated losses ranging from 10 to 60 million dollars annually. Unfortunately, scouting for stink bug damage in the field is laborious and excessively time consuming. To improve scouting accuracy and efficiency, we investigated fluorescence changes in cotton boll tissues as a result of stink bug feeding.

**Results:**

Fluorescent imaging under long-wave ultraviolet light showed that stink bug-damaged lint, the inner carpal wall, and the outside of the boll emitted strong blue-green fluorescence in a circular region near the puncture wound, whereas undamaged tissue emissions occurred at different wavelengths; the much weaker emission of undamaged tissue was dominated by chlorophyll fluorescence. We further characterized the optimum emission and excitation spectra to distinguish between stink bug damaged bolls from undamaged bolls.

**Conclusions:**

The observed characteristic fluorescence peaks associated with stink bug damage give rise to a fluorescence-based method to rapidly distinguish between undamaged and stink bug damaged cotton bolls. Based on the fluorescent fingerprint, we envision a fluorescence reflectance imaging or a fluorescence ratiometric device to assist pest management professionals with rapidly determining the extent of stink bug damage in a cotton field.

## Background

Phytophagous stink bugs (Hemiptera: Pentatomidae) comprise a critically important insect pest complex affecting worldwide food and fiber production. This group of closely related genera has a wide host range that includes fruit, vegetable, nut, fiber, and cereals in addition to numerous wild hosts [1]. Preferential feeding sites are confined to the fruiting structures [2,3], but some species feed on vegetative plant parts when fruiting structures are not available. Stink bugs have piercing/sucking mouthparts, and generalized feeding symptoms include abortion of young fruits, a predisposition to colonization by decay organisms, and cosmetic deformities. In southeastern US cotton production, feeding by stink bugs causes boll abscission, lint staining, reduced lint quality, and reduced yields [4-8]. More recent work has shown that the southern green stink bug, *Nezara viridula *(L.) (Hemiptera: Pentatomidae), is a competent vector of bacterial pathogens that causes seed and lint necrosis [9]. Stink bug damage to the 2007 southeastern cotton crop was estimated at 11.6 million dollars [10].

The scientific basis for implementation of Integrated Pest Management or IPM [11] is that insect pest populations must be monitored during periods of plant susceptibility to make cost-effective decisions about pest management. The decision to intervene (i.e. make an insecticide application) should be based on a cost/benefit analysis: expected damage attributed to the insect population versus the cost of the insecticide application [12]. Grower profits will be marginalized if the insect sampling procedure does not accurately represent the true insect density. For example, excessive spraying costs would result when damage estimates exceed the actual population density. Likewise, when the pest population is underestimated a portion of the producer's profits would be mitigated because the pests inflict excessive damage to the crop. Development of an effective sampling plan is the single most critical piece of information in the decision making process [13]. However, development of an effective sampling plan cannot proceed without a rapid and accurate sampling method. Sampling for stink bugs and their associated damage in cotton fields is time-consuming, because the bugs are aggregated and the damage is often obscured on the outside of the boll. In cotton, the most reliable characteristic is to collect quarter-sized soft bolls and dissect them for internal feeding symptoms including punctures and warty growths on the inner boll wall, lint staining, and rotten locks. Toews et al. [14] compared traditional sampling methods for stink bugs including 50 sweeps with a 38.1 cm sweep net, 3.7-linear meters of row shaken over a white drop cloth, and internal examination of 20 quarter-sized bolls. Results show that internal examination was more than 10-fold more sensitive, but required more than seven minutes per sample set of 20 bolls compared to 97 seconds and 67 seconds for the sweep net and drop cloth, respectively. In detail, the time to collect 20 bolls (123.7 ± 1.6 seconds) and examine the same 20 bolls (445.0 ± 5.3 seconds) using the current internal detection method leads to an examination time of approximately 30 seconds per boll. Moreover, a large number of bolls is required for statistical accuracy. Reay-Jones *et al. *[15] concluded that to obtain an estimate within 10% of the mean when there was 14.8% boll injury would require 112 samples of 20 bolls per sample (a total of 2240 bolls). Clearly, a new method that would reduce the examination time per boll is needed. In fact, recent efforts have been made towards characterizing changes in the production of volatile components by the cotton plant as a function of stink bug feeding [16,17].

We report here the observation of an unusual and strong fluorescent emission in cotton boll tissue that has been damaged by stink bug feeding. The objectives of this study were (1) to investigate differences in fluorescent emission between stink bug damaged and undamaged cotton bolls, (2) to find the optimum excitation and emission wavelength ranges of both stink bug-related auto fluorescence and normal tissue auto fluorescence of cotton bolls, and (3) to characterize the potential of fluorescence measurements to differentiate undamaged and damaged cotton boll tissue.

## Results and Discussion

### Visual examination

**Figure 1:**
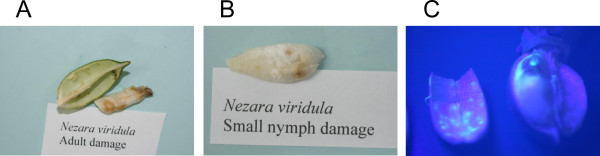
**Illustration of the damaged cotton boll**. Stink bug induced lesions appeared as yellowish-white swollen protuberances on the internal carpal wall (Panel **A**), yellow to brown staining on the lint (Panel **B**) and fluorescence reflectance imaging (Panel **C**) of the same damaged parts of the cotton boll.

In the damaged cotton boll, the symptoms of stink bug feeding on the interior boll wall included yellowish white swollen protuberances and yellowish staining on the lint (Figure 1A and 1B). Under long wavelength ultraviolet (UV) illumination, the lesion tissue and stained lint exhibited strong blue-green fluorescence (Figure 1C). The fluorescence was restricted to small circular areas centered on the spot where the stink bug mouthparts penetrated the inner carpal wall, whereas the spots in the lint were of variable size. This blue-green fluorescence was evident to the unaided eye without any special equipment other than the long-wave UV illumination.

**Figure 2:**
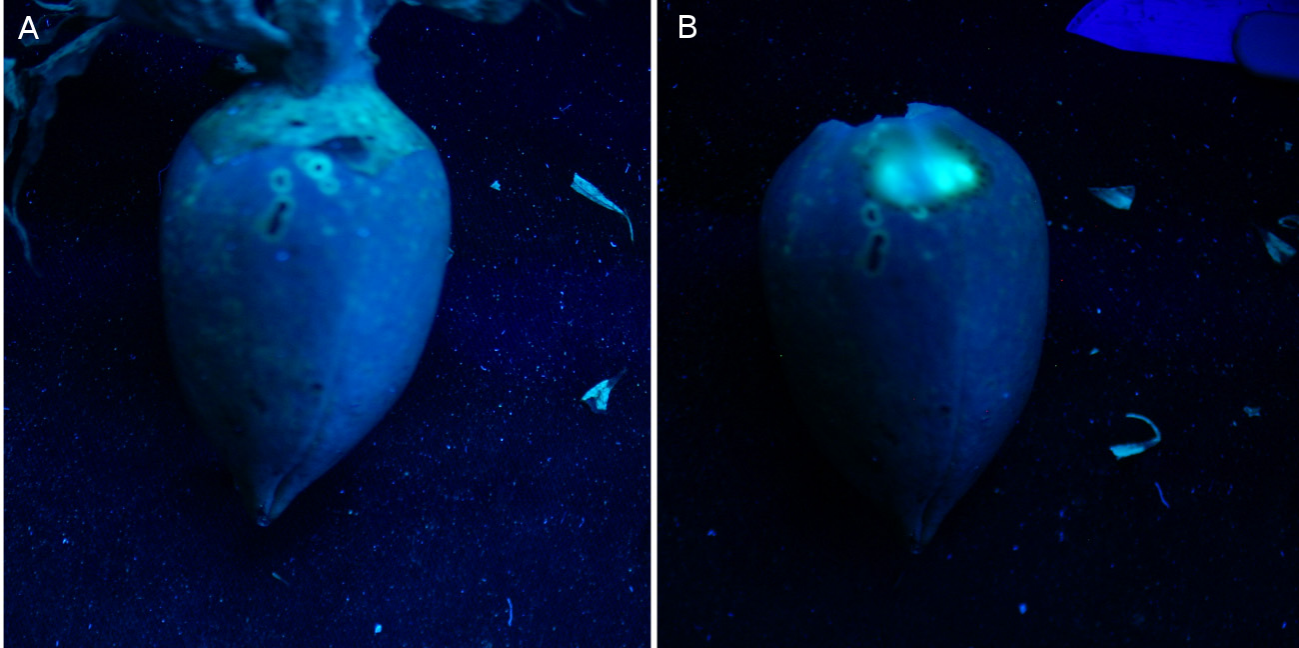
**Fluorescence reflectance imaging of stink bug damaged cotton bolls with long wavelength UV lamp illumination**. The circular blue-green spots in Panels **A **and **B **are the stink bug puncture wounds. The piercing holes are visible on some spots while invisible on some other spots. In Panel **B**, the damaged top tissue layers were removed over the circular blue-green spots.

Examination of the exterior carpal wall of the damaged cotton bolls under long-wave UV illumination exhibited similarly prominent fluorescent spots. As shown in Figure 2A-B, stink bug damaged spots exhibit strong circular blue-green fluorescence with variable sizes. Among these fluorescence spots, some have a stink bug piercing hole in the center, while others only showed the fluorescence without any piercing traces. The removal of the outer tissue layers directly over the site of the fluorescence spots revealed the fluorescence of increasing size and intensity (Figure 2B). Moreover, cutting the carpal wall along a piercing hole revealed that lesion fluorescence existed all through the thickness of the carpal wall.

**Figure 3:**
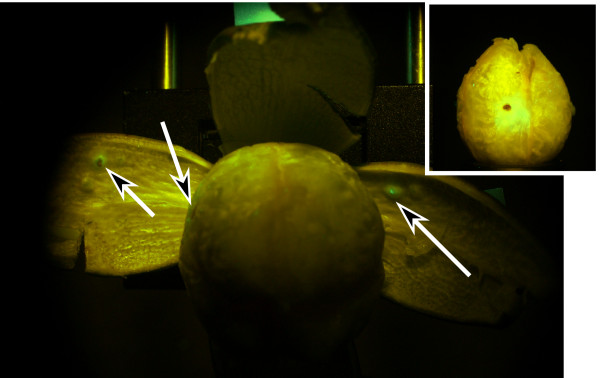
**Fluorescence reflectance imaging of a representative cotton boll that has been pierced with a sterile 31-gauge needle**. The arrows point towards the needle marks on the inner carpal wall, with one arrow pointing at the corresponding section of the lint. The lint shows the brown discoloration and the fluorescence (inset).

To elucidate the origin of the characteristic fluorescence, we punctured a boll with a sterile needle and examined its appearance (lint, warts) and its fluorescence after 48 hours. We found lint staining and the fluorescence emission described above associated with needle punctures (Figure 3). This finding excludes some possible sources of autofluorescence, such as residues - salivary exudates or bacterial contamination - introduced by the piercing mouthparts of the stinkbug. Rather, we noticed that the piercing wounds heal after 1-2 days, and that the characteristic fluorescence emission appeared to originate from the newly grown scar tissue. We submit that this observation does not reduce the significance of our findings, because there are very few insects, for example, the leaffooted bug, *Leptoglossus phyllopus *(L.) (Hemiptera: Coreidae), that pierce the cotton boll in the manner of the stink bug. However, the important point is that the specific damage by piercing/sucking is reliably detected, and this consideration applies equally to any piercing insect. We also submit, however, that none of these species are abundant at levels in cotton that are economically important. For example, there are no recommendations for treating any piercing sucking pests except stink bugs in the Pest Management Handbooks for Alabama, Georgia, North Carolina, or South Carolina.

In light of the observation of fluorescence related to needle punctures, the question of specificity arises. It is conceivable that compression causes fluorescence emission with a similar spectrum to the fluorescent spots seen around piercing wounds. The main difference is the intensity, which is higher around piercing wounds, and the shape. Piercing wounds under fluorescent imaging are round and have a distinct black center (see Figures 2 and 3). Shallow scratch marks are elongated. Suitable image analysis methods are available that can eliminate those false-positives. Furthermore, cotton bolls develop on a peduncle from the extra-axillary bud at the base of the cotton leaf petiole [18]. Although it is possible that one boll could physically contact a close neighboring boll during extreme weather conditions, the stiff peduncle and bracts would preclude penetration of the boll wall tissues and symptoms observed with stink bug feeding. Similarly, boll trauma from agricultural machinery would include boll abrasion and crushing as opposed to puncturing.

### Epifluorescence imaging

**Figure 4:**
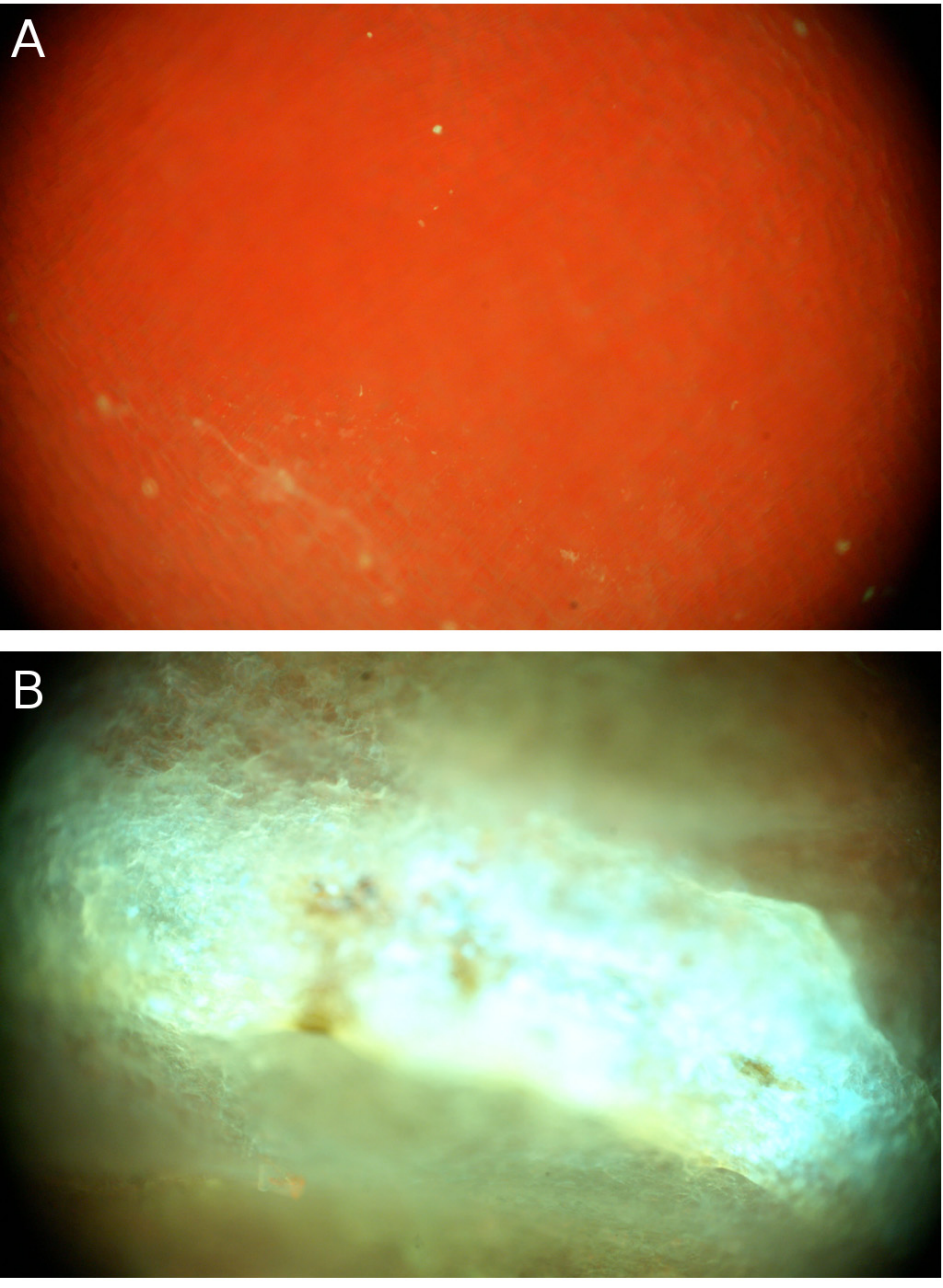
**Microscopic images of the inner carpal wall**. The figure shows epifluorescence microscope images of the inner carpal wall of an intact cotton boll (Panel **A**) and a damaged cotton boll (Panel **B**). Red chlorophyll emission dominates the intact boll, whereas the damaged boll predominantly exhibits the blue-green fluorescence.

**Figure 5:**
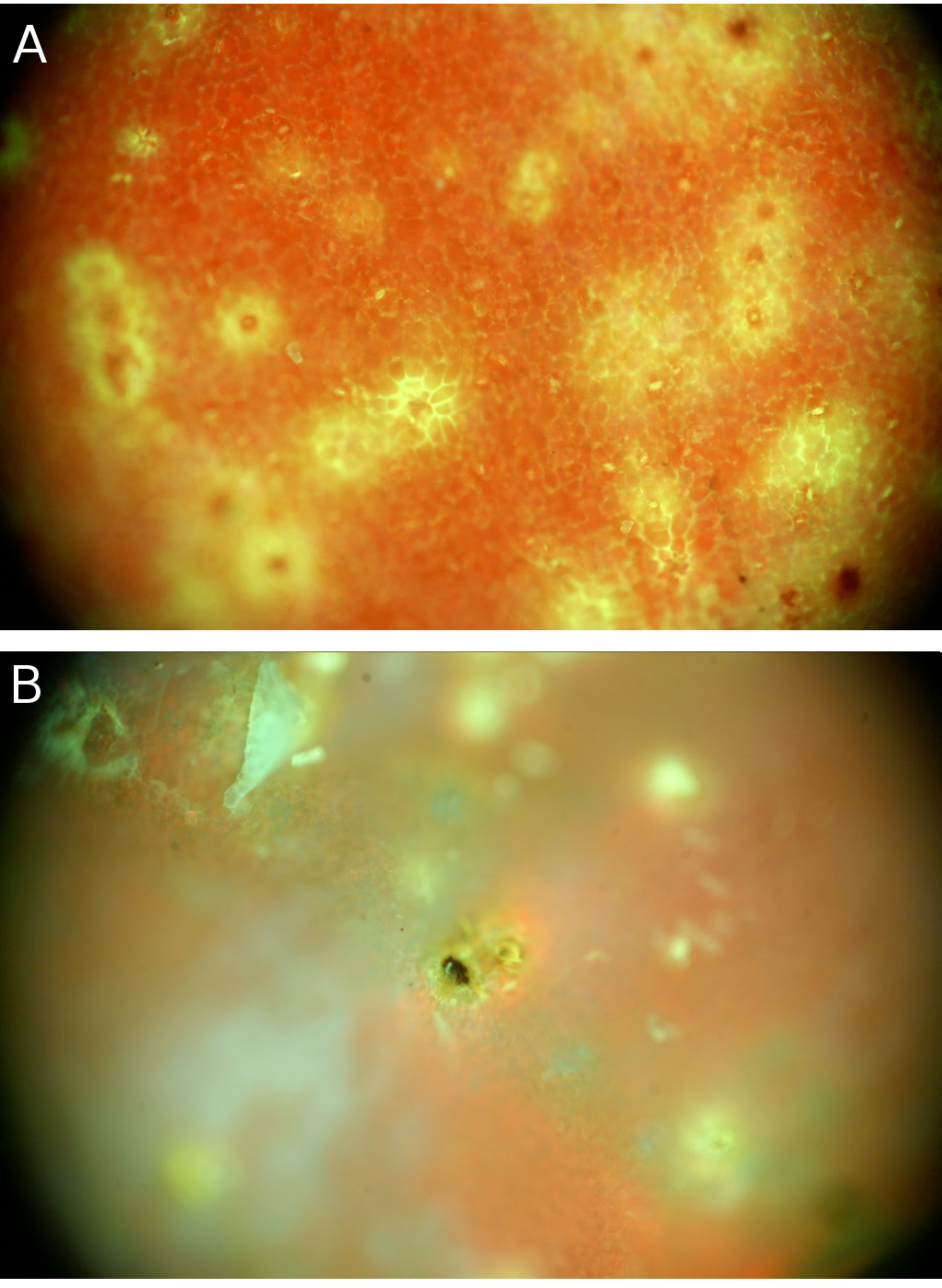
**Microscopic images of the outer carpal wall**. The figure shows epifluorescence microscope images of the outer carpal wall of an intact control cotton boll (Panel **A**) and a stink bug-damaged cotton boll (Panel **B**). Red chlorophyll emission dominates the intact boll, although small patches of reduced chlorophyll emission are present. The damaged boll (the insect puncture site can clearly be seen) shows receding red chlorophyll emission in the presence of the characteristic blue-green component.

Examination of these undamaged and stink bug damaged boll tissues under epifluorescence microscopy revealed more subtle differences in fluorescence. To facilitate microscopic examination, cotton boll walls were trimmed to ~1 cm by 1 cm sections. Undamaged interior boll tissues (Figure 4A) were dominated by red chlorophyll fluorescence, which appeared diffuse because it had been scattered by the carpal wall cell layer. The bright appearance of the red chlorophyll fluorescence is due to longer exposure times. In comparison, stink bug damaged interior boll tissues (Figure 4B) were dominated by blue-green fluorescence in the vicinity of the insect feeding puncture. Exterior boll wall imaging showed similar patterns, but the differences were more subtle. Again, in the non-damaged boll wall tissue the image was dominated by red chlorophyll emission (Figure 5A), whereas the damaged boll wall was dominated by blue-green emission surrounded by receding chlorophyll emission (Figure 5B). The apparent absence of chlorophyll (or at least its characteristic fluorescence near the feeding site) may provide an alternate approach for ratiometric spectroscopic measurement when trying to rapidly differentiate among damaged and non-damaged bolls.

### Spectral Analysis

**Figure 6:**
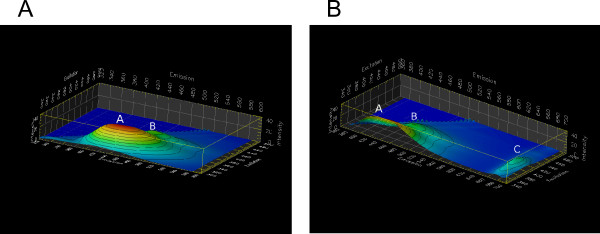
**Matrix-scan 3D graphs of stink bug induced cotton boll lesion tissues**. Panel **A**: Emission ranged from 320 nm to 585 nm while excitation ranged from 300 nm to 500 nm. The individual peak labeled A emitted from 380 nm to 480 nm with excitation from 320 nm to 380 nm, the peak labeled B emitted from 460 nm to 480 nm with excitation from 380 nm to 440 nm. Panel **B**: Emission ranged from 370 nm to 685 nm while excitation ranged from 350 nm to 500 nm. Both fluorescent peaks labeled A and B correspond to A and B in figure Panel **A**, respectively. The new peak labeled C emitted from 660 nm to 690 nm with excitation 380 nm to 440 nm.

The spectral scans for all samples were split into two separate scans to avoid artifacts from second-order diffraction in the monochromators. In the first scan, the range of excitation wavelength was set between 300 nm and 500 nm, and the emission range was between 320 nm and 585 nm. In the second scan, the excitation wavelength was between 350 nm and 500 nm and the emission range was set between 370 nm and 685 nm. The purpose of the two scans was to cover emission wavelength range that could include all the interesting characteristic fluorescent emission peaks, while avoiding the second order diffraction peak of the excitation light (i.e. a doubling of the monochromator wavelength associated with wide wavelength ranges). The matrix-scan 3D graphs of the stink bug damaged boll tissues are shown in Figure 6. The most prominent features of the spectra were a dome-shaped high-intensity plateau labeled A and a low intensity ridge labeled B. Peak wavelengths for the A-dome were 340 nm for excitation and 430 nm for emission, and peak wavelengths for the B-ridge were 410 nm for excitation and 470 nm for emission. The highest peak ratio of these two emissions *I*(*A*) = *I*(*B*) was about 3.8. The strong and relatively broad-band emission of the A-dome in these data is primarily responsible for the visually observed blue-green fluorescence in UV-excited samples. Compared to the blue-green emission in damaged tissue, the chlorophyll emission in damaged tissue marked by C (Figure 6B) was markedly weaker (by a factor of 2.6) than the A-dome and peaked at 670 nm with the similar excitation range as the B emission (excited at 380 to 440 nm). The optimum excitation wavelength for chlorophyll is near 430 nm and its emission maximum is known to be near 680 nm [19]. Their emission ratio of *I*(*B*) = *I*(*C*) was 1.3.

**Figure 7:**
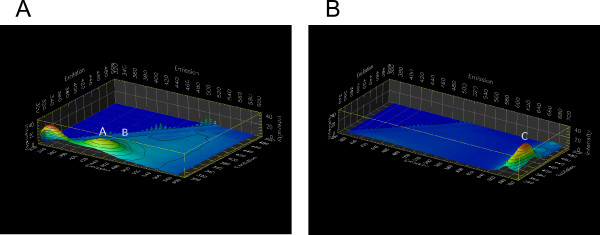
**Matrix-scan 3D graphs for undamaged boll tissue**. Panel **A**: Emission ranged from 320 nm to 585 nm and excitation ranged from 300 nm to 500 nm. The peak marked A emitted from 320 nm to 360 nm with excitation from 300 nm to 320 nm. The peak marked B emitted from 380 nm to 440 nm with excitation from 320 nm to 360 nm. Panel **B**: Emission ranged from 370 nm to 685 nm and excitation ranged from 350 nm to 500 nm. The peaks marked A and B seen in in Panel **A **were not included in Panel **B**, because the excitation range for Peaks A and B was not included. The peak marked C was for chlorophyll, which emitted from 660 nm to 690 nm with excitation 380 nm to 430 nm.

Fluorescence emission from undamaged cotton boll tissues are shown in Figure 7 as a negative control. The dome-shaped region A, corresponding to the similarly-labeled area in Figure 6, is still visible, but by a factor of 3 less intense than in the damaged tissue. The B-ridge was not discernible as a separate fluorescence peak. Chlorophyll emission (Figure 7B) exhibits a markedly stronger relative intensity than in Figure 6B. This comparison demonstrates how much the emission in the blue-green range differs between damaged and undamaged boll tissue. In fact, we found an emission ratio of *I*(*B*)/*I*(*C*) = 56 in undamaged boll tissue.

Furthermore, a new peak (unlabeled) at *λ_ex _*= 320 nm and *λ_em _*= 430 nm became evident, which was insignificant in its intensity compared to the A-dome in Figure 6A. This wavelength range is typical for protein autofluorescence [20].

The solvent, i.e., pure 70% spectroscopy-grade ethanol was also scanned in the same way to examine potential solvent background emission. Although the solvent also emitted some very weak blue-green fluorescence (~340 nm to 440 nm), it was excited by shorter wavelengths ranging from 300 nm through 320 nm and was therefore easily distinguishable from the tissue peaks. Furthermore, there was no ethanol emission when the excitation wavelength was longer than 320 nm. We conclude that the ethanol solvent does not affect the fluorescence detection of the relevant tissue components and was a suitable solvent for this study.

### Comparison of Detection Accuracy with Fluorescence and with Conventional Inspection

Bolls used for detection had a mean diameter of 24 ± 3 mm (mean ± SEM). Images of the exterior boll wall were taken under UV illumination and examined for the presence of fluorescent marks. The cotton bolls were then opened and visually examined in daylight for lint staining, puncture marks, and warts (conventional method). Across all measurements, the fluorescent detection method had a higher accuracy (greater than 90%) than the visual inspection (75% accuracy). The false positive rate of the fluorescent method (7.7%) was balanced by a false-negative rate of 6.7% (Table 1). False-negative determination with the fluorescence-based method occurred only in the recently damaged bolls (2 to 3 days before imaging) compared to a systematic bias, regardless of time since damage, when the visual inspection method was used. However, the number of false-negatives in the fluorescence-based detection group is too small to allow conclusions how the fluorescence develops over time.

**Table 1:** Accuracy and error rates for detecting damaged cotton bolls as a result of stink bug feeding.

Time since damage (d)	Visual Inspection (number bolls/total bolls)			Fluorescent Detection (number bolls/total bolls)		
	**Accuracy**	**False Positive**	**False Negative**	**Accuracy**	**False Positive**	**False Negative**
	
0	19/26	7/26	--	24/26	2/26	--
1	3/4	--	1/4	4/4	--	--
2	5/7	--	2/7	6/7	--	1/7
3	5/6	--	1/6	5/6	--	1/6
4	3/4	--	1/4	4/4	--	--
6	4/5	--	1/5	5/5	--	--
7	3/4	--	1/4	4/4	--	--

Sum (means)	42/56 (75.0%)	7/26 (26.9%)	7/30 (23.3%)	52/56 (92.9%)	2/26 (7.7%)	2/30 (6.7%)

Accuracy is defined as the sum of true positives and true negatives. Visual inspection refers to cracking the boll open and examining the interior for lint staining, puncture marks, and warts. Fluorescent detection refers to the examination of UV-illuminated fluorescent images of the exterior boll.

## Conclusions

Fluorescence spectroscopy based methods have been widely used in food and agricultural produce quality assessment and constituent identification [21-23]. Imaging methods that use either fluorescent staining or auto fluorescence can be used to visualize key constituents of the target object and have the potential to provide superior image contrast. For example, Kuensting *et al. *[24] used an autofluorescence imaging method to visualize and highlight the internal structural details in soybeans. By using a fluorescence staining imaging method, Ogawa *et al. *[25] developed a fluorescence-based technique to visualize the three-dimensional distribution of constituents in rice grains. Herein, we report a measurable and visible fluorescence emission associated with stink bug feeding of cotton bolls.

Both fluorescence reflectance imaging and epifluorescence microscopic examination conducted in this study indicated that the piercing action that is associated with stink bug feeding on cotton bolls produced a characteristic blue-green fluorescence when excited by long-wave UV exposure. Red emission from chlorophyll recedes at the same time (most prominently seen in Figure 5B). This fluorescence is unusually strong in the inner carpal wall and in affected lint, but it can also be detected from the exterior of the cotton boll as shown in Figure 5.

Our spectral analysis shows that the characteristic fluorescence is not only unique with respect to its bright intensity, but also with respect to its wavelength. Most prominently, the fluorescence emission peak at 420 nm with an excitation of 350 nm (marked with the letter A in Figure 6) differs strongly between intact boll tissue and pierced boll tissue. Moreover, we observed receding chlorophyll emission. In fact, it appears as if the intensity ratio *I*(*λ *= 420*nm*) = *I*(*λ *= 680*nm*) at an excitation of near 350 nm could serve as an indicator for the presence of piercing damage.

This observation gives rise to possible detection instruments. The ideal excitation wavelength is near the emission maxima of solid-state UV lasers and high-power UV light-emitting diodes (both 365 nm). A dual photodiode - ideally an avalanche photodiode for its higher sensitivity - would serve as the detection element. One photodiode would be sensitized with a bandpass filter for 420 nm, and the other would measure chlorophyll emission at 680 nm. The entire assembly could be housed in a wand to be used in the field. Alternatively, a CCD or CMOS imaging element would acquire fluorescence from a larger area of an individual boll. One challenge is the suppression of environmental light. Here, the detection or imaging element could be inserted into an enclosure that reduces environmental light, and further increase of the sensitivity can be achieved by employing the lock-in principle. In the field, a cotton boll would be inserted into the box for measurement. Likely, there would be no need to pluck the boll from the plant. Since fluorescence measurements take fractions of a second, an enormous time advantage could be gained over the manual examination of bolls that involves breaking bolls open.

## Methods

### Development of damaged cotton bolls

Stink bug feeding damage to cotton bolls was created using 5th instars of the southern green stink bug, *Nezara viridula *(L.) (Hemiptera: Pentatomidae). The insect colony was founded with ~ 50 adults collected from Tift County, Georgia in April 2007. The resulting colony was maintained in the lab following the methods of Harris and Todd [26] on fresh green beans, shelled green peanuts, and field corn. Adults were maintained in 37.9-liter glass aquaria while immatures were held in ventilated Petri dishes and small plastic dishes (part no. JSS16-89PP, Olcott Plastics, St. Chas, IL) at 25.0°C and 65% relative humidity. Previous research [26] suggested that these colonies may decline in vigor and viability so additional feral individuals were introduced annually.

Damaged cotton bolls were generated by caging immature stink bugs on cotton bolls of a known age in the greenhouse. Since manual examination of the fiber quality is destructive, no additional samples were grown in parallel to maturity to test for fiber quality. Briefly, picker cotton (FM 9063 B2F) was grown in a greenhouse maintained at 21 to 35°C with a 14:10 (L:D) photoperiod. Individual seeds were sown in 11.35-liter plastic pots filled with Metro Mix 300 growing medium (Sun Gro Horticulture, Bellevue, WA) and fertilized bimonthly with Osmocote 14-14-14 and Micromax 90505 (The Scotts Co. LLC, Marsville, OH). Following the methodology of Bundy et al. [27], individual white flowers were tagged daily and the subsequent bolls were allowed to develop normally for a period of 10-14 days. Then, a 30 cm long by 20 cm wide sleeve cage, containing three fifth instar stink bugs (treatment), was tightly sealed around the boll and subtending leaf for 72 hours. The age of the bolls at harvest was based on external boll diameter, and generally these bolls were 13 to 17 days after white flower. Bolls were excised from the plant immediately after exposure to the stink bugs, removed from the bag, and brought into the laboratory for examination. Undamaged bolls were prepared exactly as described above except that no insects were introduced into the sleeve cages.

To further examine the origin of the fluorescence emission, we used bolls that were not exposed to stink bugs and punctured them with a sterile syringe needle (31 Ga, 8 mm long, Beckton-Dickinson, product no. 328418). Puncturing was done manually, and care was taken that the needle penetrated into the lint tissue. The punctured bolls were either harvested immediately, or kept on the plant for 1, 2, 3, 4, 6, or 7 days after puncture before being harvested. Bolls with needle punctures were processed in the same manner as the other bolls.

### UV imaging

**Figure 8:**
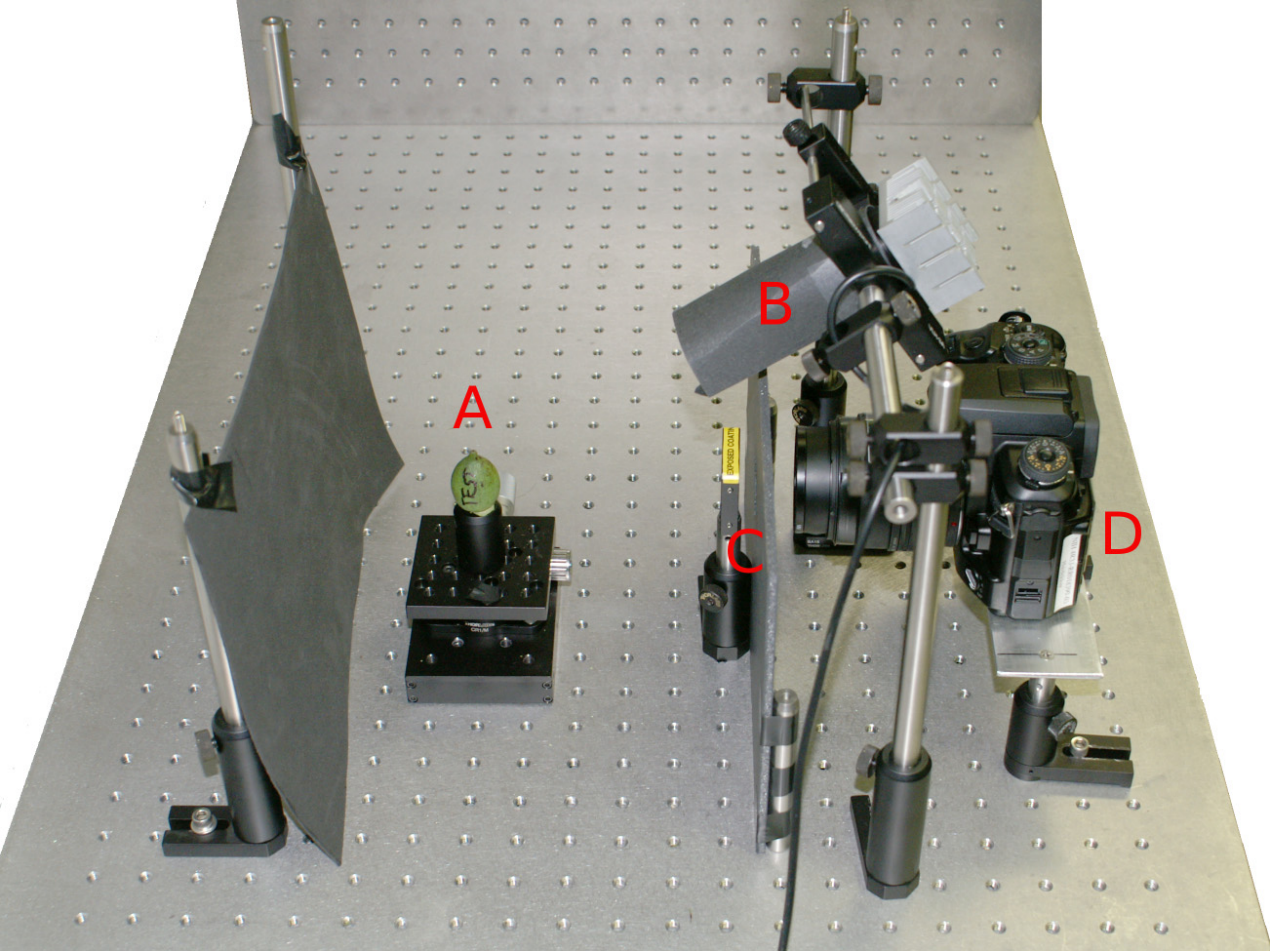
**Apparatus for fluorescent imaging of cotton bolls**. The sample is placed on a rotary stage (A) to allow the acquisition of multiple images to cover the entire surface. A light-emitting diode array (B) provides ultraviolet excitation light. Fluorescent emission passes through a longpass filter (C) that blocks UV light and reaches the SLR camera (D).

Cotton bolls were manually opened (i.e., cracked by hand without the aid of tools) and the tissues were illuminated with a 115 V/22 W long-wavelength ultraviolet lamp (Model 1925, Burton Medi-Quip Co, Van Nuys, CA) for visual inspection. To prevent UV-induced background fluorescence, the imaging table was covered with a piece of black non-fluorescent cloth. The UV source used for imaging, a high-intensity LED array (Edmund Optics NT59-369, center wavelength 370 nm, driver current 500 mA) was positioned approximately 15 cm from the sample before imaging with a standard digital SLR camera (Maxxum 7D, Konica Minolta Holdings Inc., Tokyo, Japan) equipped with a standard 50 mm fixed-focus lens and a 420 nm long-pass optical filter (Omega Optical, Brattleboro, VT). Images were taken at manual setting with an exposure time of 3s, aperture f/8 and ISO 400 sensitivity setting. The imaging apparatus is shown in Figure 8.

To examine reflectance at the microscopic level, epifluorescent imaging was conducted by using a compound microscope (Olympus IX-71) with a 10× objective (total magnification 100×) and the Deep Blue filter set, which has an excitation wavelength range in the violet with a peak at 405 nm.

Epifluorescence images were taken in an identical fashion for normal and lesion tissue of cotton bolls.

### Spectral analysis

Optimal excitation and emission wavelengths of undamaged and damaged cotton boll tissue were determined through spectral analysis. An analytical spectrofluorometer (FluoroMax-3, Horiba Jobin Yvon, Edison, NJ, USA) was used to analyze tissue spectral properties. The capabilities of the instrument include emission scanning where the selected excitation wavelength is held constant while the emission intensity is obtained as a function of the wavelength, excitation scanning where the emission wavelength is kept fixed while emission intensity is obtained as a function of excitation wavelength, and a matrix scan where fluorescence intensity is determined as a two-dimensional function of excitation and emission wavelength. The matrix scan allows to exhaustively characterize fluorescent properties of an unknown material. The result of a matrix scan is generally represented in 3-dimensional space with one fluorescence intensity axis, one excitation wavelength axis, and one emission wavelength axis.

Small solid tissue samples from the interior boll walls of *n *= 3 bolls were excised under a dissecting microscope while the subject was illuminated under long-wave UV. Only strongly fluorescing tissue was cut from the treated bolls, while similar masses of undamaged tissues were also excised from undamaged bolls for comparison. A total of 5 mg of fluorescent and non-damaged tissues were pooled separately for analyses. A glass cuvette was used to contain the sample for spectral measurement. Samples were soaked in ~5 ml of 70% spectroscopic-grade ethanol (Sigma-Aldrich, St. Louis, MO) for 48 h to extract the fluorescent materials and then 3 ml of the resulting solution were examined with the spectrofluorometer. Matrix scan 3-D graphs were created from the scan data with standard surface-rendering techniques [28].

### Comparison of Detection Accuracy with Fluorescence and with Conventional Inspection

To determine the potential for using fluorescence as a method for detecting stink bug damage, we acquired fluorescent images of 56 bolls as described above. Images were examined by a trained technician on a computer monitor. The technician had no knowledge about the treatment (control *versus *infested). Subsequently, the bolls were opened and examined for visible damage (lint staining, warts, puncture marks) [14]. We define detection accuracy as the sum of true-positives and true-negatives relative to the total number of bolls. To observe the development of the fluorescent regions over time, 26 bolls were examined within less than one day after exposure to stink bugs, and 30 bolls were kept on the plant for up to 7 days before harvesting and subsequent imaging.

## Competing interests

The authors declare that they have no competing interests.

## Authors' contributions

MAH and MT made the original discovery of stinkbug-related fluorescence and performed the initial characterization. MAH designed the imaging apparatus. JX and AM acquired all photographic and microscopic images, and JX prepared the matrix scans. All authors contributed to the data analysis and to the manuscript.
